# Dynamic Stiffness Matrix Approach to Free Vibration Analysis of Functionally Graded Rotor Bearing System Subjected to Thermal Gradients

**DOI:** 10.3390/ma15041540

**Published:** 2022-02-18

**Authors:** Bharath Obalareddy, Prabhakar Sathujoda, Roberto Citarella

**Affiliations:** 1 Department of Mechanical Engineering, Bennett University, Greater Noida 201310, India; bo2559@bennett.edu.in; 2 Department of Industrial Engineering, University of Salerno, 84084 Fisciano, Italy; rcitarella@unisa.it

**Keywords:** dynamic stiffness matrix, rotor bearing system, free vibration, functionally graded materials, non-linear temperature distribution, Wittrick–William algorithm

## Abstract

The dynamic stiffness matrix (DSM) method, an analytical method that provides exact solutions, has been used for the first time for the free vibration analysis of a functionally graded (FG) rotor bearing system subjected to temperature gradients and to investigate its application to FG rotors. The material gradation occurs based on the power law between the inner metal core and the outer ceramic rich layer of the FG rotor. The temperature gradation follows the Fourier law of heat conduction which leads to non-linear temperature distribution (NLTD) in the radial direction of the FG rotor. The development of the DSM formulations for Timoshenko FG rotor elements using the governing equations derived from translational and rotational equilibrium conditions is the novelty of the present work. The DSM of the FG rotor elements, rigid disk and linear isotropic bearings are assembled to obtain the global dynamic stiffness matrix of the FG rotor bearing system. The natural whirl frequencies are computed from the global DSM using the Wittrick–William algorithm as a root searching technique. The natural and whirl frequencies are validated with the results available in the literature and the exactness of the DSM method has been exemplified.

## 1. Introduction

The DSM method is an analytical method that assumes exact shape functions, unlike other numerical methods, such as the finite element method, which assumes the polynomial shape functions, to obtain the solution for a continuous vibration problem. Thus, it retains the accuracy and exactness of the solutions obtained for a given problem. In this context, the application of the DSM method in the domain of vibration of continuous systems has been widely investigated and reported in the literature. Chen [1] developed a general dynamic stiffness matrix for the transverse vibrations of beams based on Timoshenko beam theory. Curti et al. [2] performed a dynamic analysis of homogenous rotor bearing system using the DSM method based on Rayleigh beam theory, whereas Curti et al. [3] carried out the analysis based on Timoshenko beam theory. Banerjee [4] developed the dynamic stiffness (DS) matrix for structural elements, such as beams from the governing differential equations obtained by applying various techniques, such as D’Alembert’s principle, Hamiltonian principle Newton laws and so on. Few other works on the dynamic stiffness matrix approach are available in refs [5,6].

The frequencies are obtained from the dynamic stiffness matrix by computing the roots of the matrix determinant. However, the determinant of the dynamic stiffness matrix is usually a highly irregular transcendental function of the frequency passing through zeros and varying between infinities. This results in a highly non-linear Eigen-value problem which is difficult to solve using normal root searching techniques. In such cases, the Wittrick–William algorithm is the most efficient root searching technique, which gives the number of natural frequencies under a given trial frequency. The intervals in which the natural frequencies are located can be easily estimated from the algorithm, and the frequencies between these obtained intervals can be computed without much difficulty using a simple bisection method. The method to compute the natural frequencies using the Wittrick–William algorithm has been explained in detail in refs [7,8,9].

Functionally graded materials (FGMs) are the advanced type of inhomogeneous composite materials made up of metals, ceramics and polymers in which the smooth variation of material properties can be observed in the desired direction with the variation of material composition. The disadvantages of traditional inhomogeneous composites, such as de-lamination, de-bonding and low temperature withstanding ability which arise due to the sudden variation of the material composition, are eliminated in the FGMs. The smooth variation of material properties in the FGMs increases the temperature withstanding ability and mechanical performance of the composite material. Therefore, traditional composites are replaced by FGMs in a wide range of engineering applications, such as aerospace, mechanical, biomedical, manufacturing, and so on. Based on this context, it is crucial to study the static and dynamic behavior of FGMs.

The various works based on the dynamic behavior of (FG) beams using analytical and numerical approaches have been detailed in the literature. Aydogdu& Taskin [10] analyzed the free vibration behavior of simply supported FG beams using various classical beam theories and higher order shear deformation theories. Xiang & Yang [11], using differential quadrature method derived from Lagrange interpolation polynomials, carried out free and forced vibration analysis of variable thickness laminated FG Timoshenko beam under heat conduction. Alshorbagy et al. [12] performed the free vibration analysis of the FG Euler Bernoulli beam using the finite element method. Şimşek & Reddy [13] used the modified couple stress theory to study the bending and free vibration of FG microbeams. Celebi and Tutuncu [14], using the exact plane elasticity approach, carried out a natural frequency analysis of FG beams to obtain exact natural frequencies.

Few works are reported in the literature on the natural frequency analysis of FG rotor bearing systems using the FEM based on Timoshenko beam theory (TBT) which includes the effects of translation and rotary inertia, gyroscopic moments and transverse shear deformation. The free vibration analysis of the FG rotor bearing system having hysteresis and viscous damping effects has been carried out in refs [15,16] using FEM. The free vibration behavior of thermally loaded FG rotor-bearing systems having defects, such as transverse crack [17], porosities [18] and corrosion defect [19] have been investigated, and the influence of these defects on the natural and whirl frequencies has been studied in detail in the literature.

Due to the superiority of theDSM method in terms of exactness and accuracy of the solutions obtained, compared to that of approximate/numerical methods, several works based on natural frequency analysis of FG beams using DSM formulation havebeen detailed in the literature. Based on the general approach presented by Banerjee [4] to develop the DSM formulation, Su et al. [20] developed the DSM formulation to analyze the free vibration behavior of the FG beams. Further extensions of the work are also reported in the literature by Su& Banerjee [21] and Banerjee &Ananthapuvirajah [22], which includes the analysis of mode shapes of the FG beams. The Wittrick–William algorithm has been used as a root searching technique to calculate the free vibration frequencies from the global dynamic stiffness matrix in the above-listed works based on the free vibration of FG beams. Hao et al. [23] used the Monte Carlo based simulation to study the parametric random vibration of axially moving laminated shape memory alloy. Akgöz and Civalek [24] investigated the static bending and buckling behavior of the size dependent micro-beams based on shear deformation and modified strain gradient theory. Akbas et al. [25] discussed the dynamic responses of fiberreinforced composite Timoshenko beam obtained using the Ritz method. The systems analyzed in the above works can be re-modeled using FG rotors/beams, and the DSM formulations can be developed based on the approach used in this study to investigate the advantages and disadvantages of replacing the materials used in these systems with FGMs.

Even though significant works have been reported on the use of the DSM method to investigate the free vibration analysis of FG beams, not enough attention has been paid to its applicability to vibration behavior rotor systems. A couple of works [2,3] have been reported on the formulation of the DSM method to calculate the natural frequencies of homogeneous rotor bearing systems without any thermal loading. However, to the best of the author’s knowledge, there are no works reported in the literature on the formulation and applicability of the DSM method to compute the free vibration frequencies of FG rotor systems subjected to thermal gradients. Therefore the present work mainly focuses on the application of the DSM method on free vibrations of the functionally graded rotor-bearing system.The DSM formulation has been developed based on TBT for the free vibration analysis of the FG rotor bearing system subjected to a non-linear temperature gradient. The natural and whirl frequencies of the FG rotor bearing system havebeen computed using the Wittrick–William algorithm as a root searching technique.

## 2. Material Modeling

**Figure 1:**
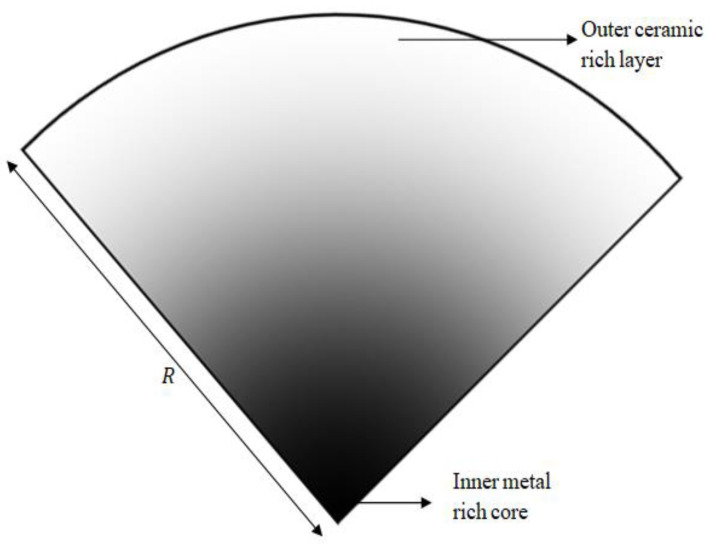
Material gradation in conical cut section of FG rotor cross-section.

The FG rotor is composed of metal and ceramic which can be classified under metal-ceramic FGMs. The material gradation of the FG rotor occurs between the inner metal-rich core and the outer ceramic-rich layer in the radial direction, as shown in Figure 1 based on power law. The volume fraction of ceramic is varied in the radial direction based on power law as:

(1) $$V_{c}\left(r\right)={\left[\frac{r-R_{i}}{R_{o}-R_{i}}\right]}^{k}$$

The relationship between the volume fractions of the metal and ceramic constituents at any given layer of the FG rotor can be written as:

(2) $$V_{m}\left(r\right)+V_{c}\left(r\right)=1$$

According to the rule of mixtures of composite materials, effective material properties P of a given layer of the FG rotor can be expressed as:

(3) $$P\left(r,T\right)=P_{m}\left(T\right)V_{m}\left(r\right)+P_{c}\left(T\right)V_{c}\left(r\right)$$

Solving the above three equations, effective material property for any given layer of the FG rotor can be obtained as:

(4) $$P\left(r,\;T\right)=P_{m}\left(T\right)+\left(P_{c}\left(T\right)-P_{m}\left(T\right)\right){\left[\frac{r-R_{i}}{R_{o}-R_{i}}\right]}^{k}$$

The FG rotor is subjected to a temperature gradient that follows one-dimensional steady-state Fourier heat conduction equation, without heat generation, expressed below.

(5) $$\frac{d}{dr}\left[K\left(r\right)\frac{dT}{dr}\right]=0$$

The boundary conditions *T* =*T_i_* at *r* = *R_i_* and *T* = *T_o_* at *r* = *R_o_* have been applied, and non-linear temperature distribution as a function of the radial distance from the centerof the rotor has been obtained by Lanhe [26], expressed as a polynomial series of seven terms given below.

(6) $$T\left(r\right)=T_{i}+\Delta T\left[\frac{\sum_{j=0}^{5}\left\{\frac{{\left(-1\right)}^{j}}{jk+1}{\left(\frac{K_{oi}}{K_{i}}\right)}^{j}{\left(\frac{r-R_{i}}{R_{o}-R_{i}}\right)}^{jk+1}\right\}}{\frac{{\left(-1\right)}^{j}}{jk+1}{\left(\frac{K_{oi}}{K_{i}}\right)}^{j}}\right]$$

Here, $K_{oi}=K_{o}-K_{i}$ and $\Delta T=T_{o}-T_{i}$. The material properties of the metal and ceramic in the FG rotor subjected to temperature gradient vary as a function of material temperature. The material property as a function of material temperature can be expressed as:

(7) $$P\left(T\right)=P_{0}\left(P_{-1}T^{-1}+1+P_{1}T+P_{2}T^{2}+P_{3}T^{3}\right)$$

where *P*_−1_*, P*_0_*, P*_1_*, P*_2_ and *P*_3_ are the temperature coefficients and are unique for a given material property of a given material. The temperature coefficients for the material properties of various materials have been listed by Reddy & Chin [27].

## 3. Methodology

**Figure 2:**
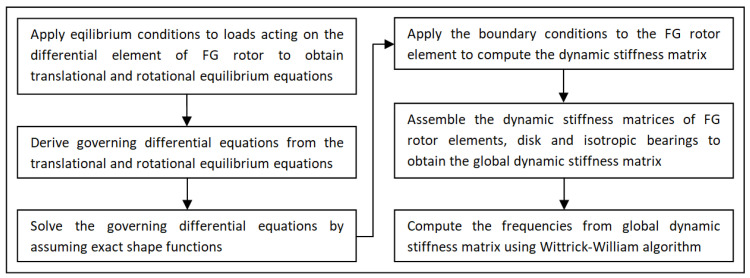
DSM Methodology flow chart for free vibration analysis of FG rotor bearing system.

The step-by-step methodology followed in the present study to develop the DSM formulation for the FG rotor and to conduct the free vibration analysis of the FG rotor bearing system is shown in Figure 2 in the form of a flow chart.To begin with, the expressions for various loads acting on the differential element of the FG rotor at the deformed equilibrium state have been considered, and the governing differential equations of motion for the FG rotor element have been derived by applying the conditions of equilibrium. The exact shape functions have been assumed to obtain the exact solution for the governing differential equations, and the dynamic stiffness matrix has been developed for the FG rotor element. The dynamic stiffness matrices of the FG rotor system, such as rotor, disc and bearings are assembled together to obtain the global dynamic stiffness matrix of the FG rotor bearing system. The Wittrick–William algorithm has been employed as a root searching technique to compute the natural and whirl frequencies from the global dynamic stiffness matrix.

## 4. DSM Formulation for FG Rotor Element

**Figure 3:**
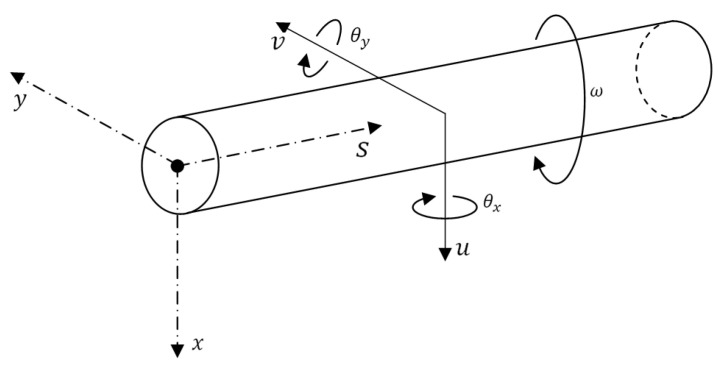
Co-ordinate system and degrees of freedom of the rotor element.

**Figure 4:**
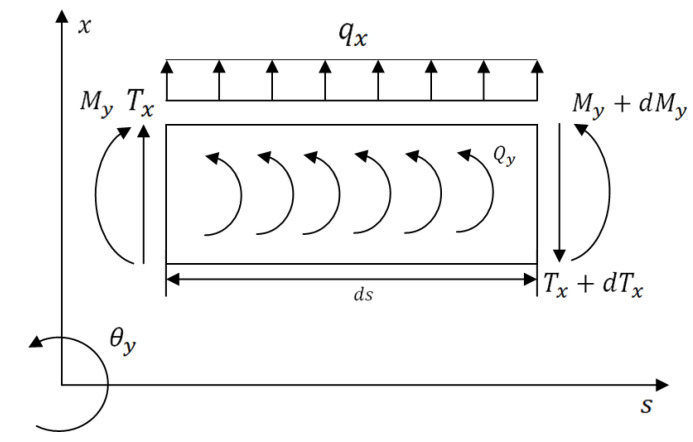
Sign conventions for loads acting on the differential rotor element in (*x*, *s*) plane.

**Figure 5:**
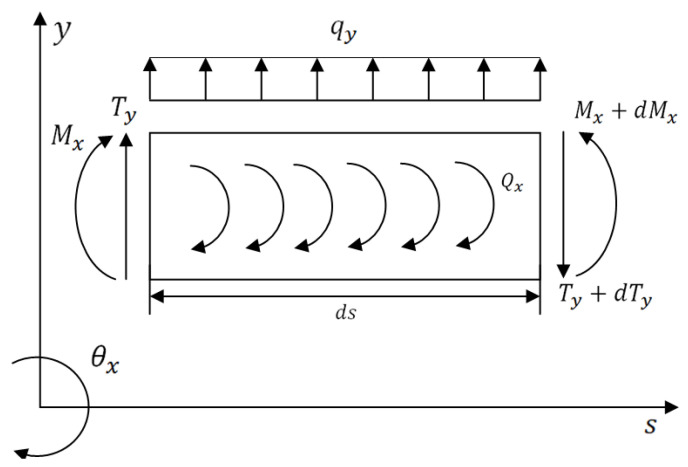
Sign conventions for loads acting on the differential rotor element in (*y*, *s*) plane.

The general coordinate system (*x*,*y*,*s*) and the nodal degrees of freedom of the FG rotor element are represented in Figure 3. The loads acting on the differential FG rotor element in (*x*,*s*) and (*y*,*s*) planes at equilibrium are represented in Figure 4 and Figure 5, respectively. The expressions for these various loads acting on (*x*,*s*) and (*y*,*s*) planes are defined based on Timoshenko beam theory which considers the effects of translation and rotation inertia, bending and gyroscopic moment, and transverse shear. The corresponding translational and rotational equilibrium conditions in both planes have been derived.

**In** (x,s) **plane:**

The expressions for various loads acting on the plane, represented in Figure 4, are listed below.

(8) $$q_{x}=-\iint \rho \left(r,T\right)dA\times \frac{d^{2}u}{dt^{2}}$$

(9) $$Q_{y}=-\iint \rho \left(r,T\right)\frac{r^{2}}{2}dA\times \frac{d^{2}\theta_{y}}{dt^{2}}+\iint \rho \left(r,T\right)r^{2}dA\times \omega \frac{d\theta_{x}}{dt}$$

(10) $$T_{x}=\iint \kappa \left(r,T\right)G\left(r,T\right)dA\times \left(\frac{du}{ds}-\theta_{y}\right)$$

(11) $$M_{y}=\iint E\left(r,T\right)\frac{r^{2}}{2}dA\times \frac{d\theta_{y}}{ds}$$

From Equations (8)–(11), the translational and rotational equilibrium conditions of the plane are obtained in Equations (12) and (13), respectively.

(12) $$\iint \kappa \left(r,T\right)G\left(r,T\right)dA\times \left(\frac{d^{2}u}{ds^{2}}-\frac{d\theta_{y}}{ds}\right)=\iint \rho \left(r,T\right)dA\times \frac{d^{2}u}{dt^{2}}$$

(13) $$\iint E\left(r,T\right)\frac{r^{2}}{2}dA\times \frac{d\theta_{y}}{ds}=\left[\begin{array}{c} -\iint \kappa \left(r,T\right)G\left(r,T\right)dA\times \left(\frac{du}{dz}-\theta_{y}\right)+\\ \iint \rho \left(r,T\right)\frac{r^{2}}{2}dA\times \frac{d^{2}\theta_{y}}{dt^{2}}-\iint \rho \left(r,T\right)r^{2}dA\times \omega \frac{d\theta_{x}}{dt} \end{array}\right]$$

**In** (y,s) **plane:**

The expressions for various loads acting on the plane, represented in Figure 5, are listed below.

(14) $$q_{y}=-\iint \rho \left(r,T\right)dA\times \frac{d^{2}v}{dt^{2}}$$

(15) $$Q_{x}=-\iint \rho \left(r,T\right)\frac{r^{2}}{2}dA\times \frac{d^{2}\theta_{x}}{dt^{2}}-\iint \rho \left(r,T\right)r^{2}dA\times \omega \frac{d\theta_{y}}{dt}$$

(16) $$T_{y}=-\iint \kappa \left(r,T\right)G\left(r,T\right)dA\times \left(\frac{dv}{ds}+\theta_{x}\right)$$

(17) $$M_{x}=-\iint E\left(r,T\right)\frac{r^{2}}{2}dA\times \frac{d\theta_{x}}{ds}$$

From the Equations (14)–(17), the translational and rotational equilibrium conditions of the plane are obtained as given below, respectively.

(18) $$\iint \kappa \left(r,T\right)G\left(r,T\right)dA\times \left(\frac{d^{2}v}{ds^{2}}+\frac{d\theta_{x}}{ds}\right)=\iint \rho \left(r,T\right)dA\times \frac{d^{2}v}{dt^{2}}$$

(19) $$\iint E\left(r,T\right)\frac{r^{2}}{2}dA\times \frac{d\theta_{x}}{ds}=\left[\begin{array}{c} \iint \kappa \left(r,T\right)G\left(r,T\right)\times \left(\frac{dv}{dz}-\theta_{x}\right)+\\ \iint \rho \left(r,T\right)\frac{r^{2}}{2}dA\times \frac{d^{2}\theta_{x}}{dt^{2}}+\iint \rho \left(r,T\right)r^{2}dA\times \omega \frac{d\theta_{y}}{dt} \end{array}\right]$$

### 4.1. Governing Differential Equations of Motion

The variables *θ_x_* and *θ_y_* are eliminated from the equilibrium condition equations of the planes (*x*,*s*) and (*y*,*s*) given in Equations (12), (13), (18)and (19), respectively. The resultant equations are combined by introducing a complex variable *z* = *x* + *iy*, to obtain the governing equation for the Timoshenko FG rotor element in terms of total deflection *z* as:

(20) $$\iint E(r,T)\frac{r^{2}}{2}dA\frac{d^{4}z}{ds^{4}}+\left[\begin{array}{l} \iint \rho (r,T)\frac{r^{2}}{2}dA\frac{\iint \rho (r,T)dA}{\iint \kappa (r,T)G(r,T)}\frac{d^{4}z}{dt^{4}}+\iint \rho (r,T)dA\frac{d^{4}z}{dt^{4}}-\\ (\iint \rho (r,T)\frac{r^{2}}{2}dA+\frac{\iint \rho (r,T)dA}{\iint \kappa (r,T)G(r,T)}\iint E(r,T)\frac{r^{2}}{2}dA)\frac{d^{4}z}{ds^{2}dt^{2}}\\ +i\omega \iint \rho (r,T)\frac{r^{2}}{2}dA(\frac{d^{3}z}{ds^{2}dt}-\frac{\iint \rho (r,T)dA}{\iint \kappa (r,T)G(r,T)}\frac{d^{3}z}{dt^{3}}) \end{array}\right]=0$$

The variables x and y are eliminated from the equilibrium condition equations of the planes (*x*,*s*) and (*y*,*s*) given in Equations (12), (13), (18) and (19), respectively. The resultant equations are combined by introducing a complex variable$\;\theta =\theta_{y}-i\theta_{x}$, to obtain the governing equation for the Timoshenko FG rotor element in terms of bending slope *θ* as:

(21) $$\iint E(r,T)\frac{r^{2}}{2}dA\frac{d^{4}\theta }{ds^{4}}+\left[\begin{array}{l} \iint \rho (r,T)\frac{r^{2}}{2}dA\frac{\iint \rho (r,T)dA}{\iint \kappa (r,T)G(r,T)}\frac{d^{4}\theta }{dt^{4}}+\iint \rho (r,T)dA\frac{d^{4}\theta }{dt^{4}}-\\ (\iint \rho (r,T)\frac{r^{2}}{2}dA+\frac{\iint \rho (r,T)dA}{\iint \kappa (r,T)G(r,T)}\iint E(r,T)\frac{r^{2}}{2}dA)\frac{d^{4}\theta }{ds^{2}dt^{2}}\\ +i\omega \iint \rho (r,T)\frac{r^{2}}{2}dA(\frac{d^{3}\theta }{ds^{2}dt}-\frac{\iint \rho (r,T)dA}{\iint \kappa (r,T)G(r,T)}\frac{d^{3}z}{dt^{3}}) \end{array}\right]=0$$

The structure and the coefficients of the governing equations of Timoshenko FG rotor element in terms of total deflection, z and bending slope, θ given in Equations (20) and (21) are the same.

### 4.2. Solution Procedure for the Governing Differential Equations

The solution for the total deflection, *z* and the bending slope, *θ* in the Equations (20) and (21) for the harmonic motion of the Timoshenko FG rotor element can be assumed as $z\left(s,t\right)=Z\left(s\right)e^{i\lambda t}$ and $\theta \left(s,t\right)=\Theta \left(s\right)e^{i\lambda t}$ where Z(s) and Θ(s) are the position-dependent amplitudes of motion. Substituting the assumed harmonic solutions in the corresponding equations, the Equations (20) and (21) can be rewritten as:

(22) $$\iint E\left(r,T\right)\frac{r^{2}}{2}dA\frac{d^{4}Z}{ds^{4}}+F\frac{d^{2}Z}{ds^{2}}+HZ=0$$

(23) $$\iint E\left(r,T\right)\frac{r^{2}}{2}dA\frac{d^{4}\Theta }{ds^{4}}+F\frac{d^{2}\Theta }{ds^{2}}+H\Theta =0$$

where,

$$F=\left[\left(\iint \rho \left(r,T\right)\frac{r^{2}}{2}dA+\frac{\iint \rho \left(r,T\right)dA}{\iint \kappa \left(r,T\right)G\left(r,T\right)}\iint E\left(r,T\right)\frac{r^{2}}{2}dA\right)\lambda^{2}-2\omega \lambda \iint \rho \left(r,T\right)\frac{r^{2}}{2}dA\right]$$

$$H=\left[\iint \rho \left(r,T\right)\frac{r^{2}}{2}dA\frac{\iint \rho \left(r,T\right)dA}{\iint \kappa \left(r,T\right)G\left(r,T\right)}\lambda^{2}\left(\lambda^{2}-2\omega \lambda \right)-\lambda^{2}\iint \rho \left(r,T\right)dA\right]$$

The solution for Equations (22) and (23) can be assumed as:

(24) $$Z\left(s\right)=e^{\sqrt{\tau }\;s/l}$$

(25) $$\Theta \left(s\right)=e^{\sqrt{\tau }\;s/l}$$

Substituting the Equations (24) and (25) in Equations (22) and (23), respectively, the resultant characteristic equations can be obtained as:

(26) $$\tau^{2}+2V\tau -W=0$$

where,

$$V=\frac{1}{2}Fl^{2}/\iint E\left(r,T\right)\frac{r^{2}}{2}dA$$

$$W=-Hl^{4}/\iint E\left(r,T\right)\frac{r^{2}}{2}dA$$

The two roots of the quadratic characteristic Equation (26) are:

$$\tau_{1}=-V+\sqrt{V^{2}+W}\tau_{2}=-V-\sqrt{V^{2}+W}$$

Considering the condition  W>0, the roots $\tau_{1}$ and $\tau_{2}$ can be written as:

(27) $$\tau_{1}=\alpha^{2},\;\tau_{2}=-\beta^{2}=>\sqrt{\tau_{1}}=\pm \alpha ,\;\sqrt{\tau_{2}}=\pm i\beta$$

From Equation (27), the solution for amplitude functions Z(s) and Θ(s) can be expressed as:

(28) $$Z\left(s\right)=C_{1}\cosh\left(\frac{\alpha s}{l}\right)+C_{2}\sinh\left(\frac{\alpha s}{l}\right)+C_{3}\cos\left(\frac{\beta s}{l}\right)+C_{4}\sin\left(\frac{\beta s}{l}\right)$$

(29) $$\Theta \left(s\right)=C_{5}\cosh\left(\frac{\alpha s}{l}\right)+C_{6}\sinh\left(\frac{\alpha s}{l}\right)+C_{7}\cos\left(\frac{\beta s}{l}\right)+C_{8}\sin\left(\frac{\beta s}{l}\right)$$

where, $C_{i}\left(i=1,\;2,\;\ldots ,\;8\right)$ are the constants of integration. 

The two sets of integration constants $\left(C_{1}\;\mathrm{to}\;C_{4}\right)$ and $\left(C_{5}\;\mathrm{to}\;C_{8}\right)$ in Equations (28) and (29), respectively are related to each other due to the coupling between the lateral deflection and the bending slope in both (*x*,*s*) and (*y*,*s*) planes given by the coupled equilibrium Equations (12), (13), (17)and (18), respectively. The relationship between the two sets of integration constants has been derived.

The either of the translational or rotational equilibrium equations of (*x*,*s*) and (*y*,*s*) planes canbe combined together and rewritten in terms of the amplitude functions *Z*(*s*) and Θ(s) to derive the relationship between the two sets of integration constants. The translational equilibrium Equations (12) and (17) of (*x*,*s*) and (*y*,*s*) planes, respectively, have been considered and the following resultant equation by applying the procedure mentioned before has been obtained.

(30) $$\iint \kappa \left(r,T\right)G\left(r,T\right)dA\times \left(\frac{d^{2}Z\left(s\right)}{ds^{2}}-\frac{d\Theta \left(s\right)}{ds}\right)=\lambda^{2}Z\left(s\right)\iint \rho \left(r,T\right)dA$$

Substituting the Equations (24) and (25) in Equation (30) above, the following relationship between the two sets of integration constant has been derived,

$$C_{6}=aC_{1},\;C_{5}=aC_{2},\;C_{7}=-bC_{4},\;C_{8}=bC_{3}$$

where,

$$a=\left(\frac{l}{\alpha }\right)\left[\frac{\lambda^{2}\iint \rho \left(r,T\right)dA}{\iint \kappa \left(r,T\right)G\left(r,T\right)dA}+{\left(\frac{\alpha }{l}\right)}^{2}\right]$$

$$b=\left(\frac{l}{\beta }\right)\left[\frac{\lambda^{2}\iint \rho \left(r,T\right)dA}{\iint \kappa \left(r,T\right)G\left(r,T\right)dA}-{\left(\frac{\beta }{l}\right)}^{2}\right]$$

### 4.3. Dynamic Stiffness Matrix Coefficients

**Figure 6:**
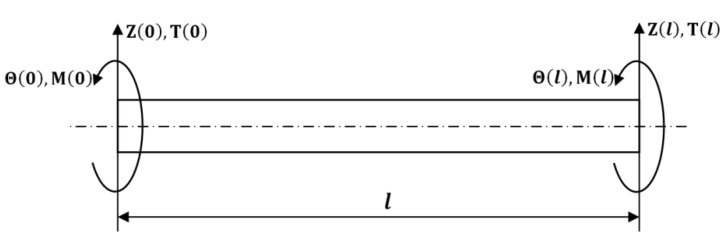
The boundary conditions at the ends of the FG rotor element.

The boundary conditions of the Timoshenko FG rotor element at its ends which includes the generalized loads and displacements are represented in Figure 6. 

The relationship between the loads and displacements of the rotor element has been written in matrix form as:

$$\left[K_{e}\right]\cdot \left\{D_{e}\right\}=\left\{F_{e}\right\}$$

(31) $$\left[\begin{array}{cc} \begin{array}{cc} K_{11} & K_{12}\\ K_{21} & K_{22} \end{array} & \begin{array}{cc} K_{13} & K_{14}\\ K_{23} & K_{24} \end{array}\\ \begin{array}{cc} K_{31} & K_{32}\\ K_{41} & K_{42} \end{array} & \begin{array}{cc} K_{33} & K_{34}\\ K_{43} & K_{44} \end{array} \end{array}\right]\left\{\begin{array}{c} \begin{array}{c} Z\left(0\right)\\ \Theta \left(0\right) \end{array}\\ \begin{array}{c} Z\left(l\right)\\ \Theta \left(l\right) \end{array} \end{array}\right\}=\left\{\begin{array}{c} \begin{array}{c} T\left(0\right)\\ -M\left(0\right) \end{array}\\ \begin{array}{c} -T\left(l\right)\\ M\left(l\right) \end{array} \end{array}\right\}$$

Here, *K* is the dynamic stiffness matrix and $K_{ij}\;\left(i,j=1,2,3,4\right)$ are the dynamic stiffness matrix coefficients of FG rotor elements. The Equations (10) and (16) are combined to obtain the expression for *T*(*s*), and the Equations (11) and (17) are combined to obtain the expression for *M*(*s*) in terms of amplitude functions as:

(32) $$T\left(s\right)=-\iint \kappa \left(r,T\right)G\left(r,T\right)dA\times \left(\frac{dZ}{ds}+\Theta \right)$$

(33) $$M\left(s\right)=\frac{1}{2}\iint E\left(r,T\right)r^{2}dA\times \frac{d\Theta }{ds}$$

The expressions for total deflection, bending slope, shear force and bending moment at the beam ends can be obtained from the Equations (28), (29), (32) and (33), respectively. Substituting these expressions in Equation (31), the expressions for the dynamic stiffness coefficients of the dynamic stiffness matrix can be derived. Looking at the expressions of dynamic stiffness matrix coefficients, the dynamic stiffness matrix appears to exhibit the asymmetric structure, the inherent property of the dynamic stiffness matrix being symmetric in nature. The symmetric structure of the dynamic stiffness matrix can be obtained by using the following identity:

(34) $$\frac{\iint \kappa \left(r,T\right)G\left(r,T\right)dA}{\iint E\left(r,T\right)r^{2}dA}=-\frac{\alpha \beta ab}{2cl^{2}}$$

In the above equation, $c=\left(\lambda^{2}\iint \rho \left(r,T\right)dA\right)/\left(\iint \kappa \left(r,T\right)G\left(r,T\right)dA\right)$. The expressions of dynamic stiffness matrix coefficients of the dynamic stiffness matrix can be written as:

$$K_{11}=K_{33}=-\frac{\iint E\left(r,T\right)r^{2}dA}{2D}ab\cdot \left(a\alpha -b\beta \right)\cdot \left(\cos\beta \sinh\alpha -\sin\beta \cosh\alpha \right)$$

$$K_{22}=K_{44}=\frac{\iint E\left(r,T\right)r^{2}dA}{2D}\left(a\alpha -b\beta \right)\cdot \left(\sin\beta \cosh\alpha -\cos\beta \sinh\alpha \right)$$

$$K_{13}=K_{31}=\frac{\iint E\left(r,T\right)r^{2}dA}{2D}ab\cdot \left(a\alpha -b\beta \right)\cdot \left(a\sinh\alpha -b\sin\beta \right)$$

$$K_{24}=K_{42}=-\frac{\iint E\left(r,T\right)r^{2}dA}{2D}\left(a\alpha -b\beta \right)\cdot \left(b\sinh\alpha +a\sin\beta \right)$$

$$K_{12}=K_{21}=-K_{34}=-K_{43}=-\frac{\iint E\left(r,T\right)r^{2}dA}{2D}ab\cdot \left[\begin{array}{c} \left(a\alpha +b\beta \right)\cdot \left(\cos\beta \cosh\alpha -1\right)\\ +\left(a\beta -b\alpha \right)\cdot \sin\beta \sinh\alpha \end{array}\right]$$

$$K_{23}=K_{32}=-K_{41}=-K_{14}=-\frac{\iint E\left(r,T\right)r^{2}dA}{2D}ab\cdot \left(a\alpha -b\beta \right)\cdot \left(\cos\beta -\cosh\alpha \right)$$

$\mathrm{where},\;D=l\cdot \left[2ab\cdot \left(\cos\beta \cosh\alpha -1\right)+\left(a^{2}-b^{2}\right)\cdot \sin\beta \sinh\alpha \right]$.

The 4 × 4 DS matrix of the FG rotor element with dynamic stiffness coefficients derived above can be used to find the natural frequencies of the FG rotor element for different boundary conditions. Any number of DS matrices of FG rotor elements of the same/different element lengths can be assembled to the required rotor length in addition to the DS matrices of uniform rigid disk and linear isotropic bearings obtained from Curti et al. [2]. The effect of the gyroscopic moment of disk rotation has been included in the DS matrix of the uniform rigid disk. The final assembled global DS matrix can be used to find the natural whirl frequencies of the FG rotor bearing system. The reader may refer to the assembly procedure detailed in Curti et al. [2] to possess a greater understanding in regard to the assembly procedure of DS matrices for a rotor bearing system.

## 5. Wittrick–William Algorithm

The Wittrick–William algorithm has been employed as a root searching technique to compute the modal frequencies from the global dynamic stiffness matrix. The algorithm gives the number of natural frequencies below the trial frequency specified by the user. Thus, the upper bounds and lower bounds for each of the modal frequencies can be estimated, and the modal frequencies between corresponding bounds can be obtained by calculating the root of the matrix determinant between the bounds using the bisection method. The number of modal frequencies $J\left(\omega^{*}\right)$ below the trial frequency $\omega^{*}$ specified by the user can be obtained as:

(35) $$J\left(\omega^{*}\right)=J_{o}+J_{k}=J_{o}+s\left\{K^{\Delta }\left(\omega^{*}\right)\right\}$$

In the equation above, $K^{\Delta }$ is the upper triangular matrix obtained through the Gaussian elimination of global dynamic stiffness matrix *K*, $J_{k}$ is the number of negative leading diagonal elements in $K^{\Delta }\left(\omega^{*}\right)$ known as sign count, which is represented as $s\left\{K^{\Delta }\left(\omega^{*}\right)\right\}$ and $J_{o}$ is the number of clamped-clamped frequencies below $\omega^{*}$ of the structural elements of the system.

## 6. Results and Discussions 

**Figure 7:**
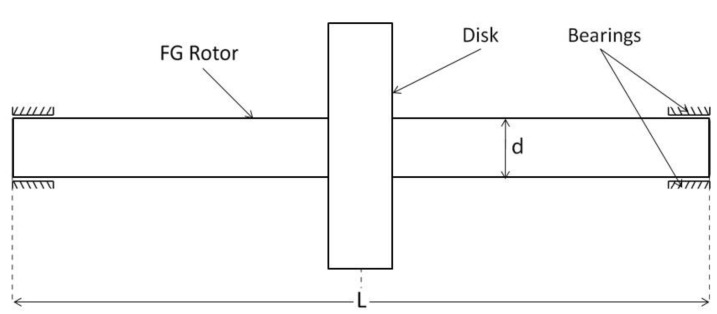
Functionally graded rotor-bearing system with a disk at the mid-span.

In the present study, the global dynamic stiffness matrix has been derived for the FG rotor bearing system in which the FG rotor is subjected to temperature gradients. The FG rotor consists of a uniform rigid disk at the centerand is supported on linear isotropic bearings at the ends as shown in Figure 7. A code has been developed in Python Programming Language (PPL) to obtain the natural and whirl frequencies of the FG rotor bearing system from its global dynamic stiffness matrix developed in this paper using the Wittrick–William algorithm. The step-by-step validation procedure of the developed DSM method with the published results has been presented in the following subsections.

### 6.1. Validation of DSM Formulation—SteelRotor Bearing System

To begin with, the natural frequencies of the steel rotor bearing system have been computed from the global DS matrix using the Wittrick–William algorithm. The following properties of the steel rotor bearing system are used to validate the results from the literature [28].

Steel material properties: $E=208\;\mathrm{GPa},\;\nu =0.3,\;\rho =7800\;\mathrm{kg}/m^{3}$.

Rotor dimensions: L=0.5 m d=0.2 cm.

Bearing stiffness, $k_{b}=10^{5}\;N/m$.

Disk properties: $M_{d}=5.5\;\mathrm{kkg}\;I_{d}=0.00773\;\mathrm{kg}\cdot m^{2}\;I_{p}=0.01546\;\mathrm{kg}\cdot m^{2}$.

The natural frequencies obtained from DSM are compared with FEM results and tabulated in Table 1.The results obtained are in good agreement with the literature. The approximate mode shapes and corresponding natural frequencies obtained from the FEM method varyfrom the exact solutions obtained from the DSM method at higher modes compared to lower modes. Therefore, there is a higher variation in the natural frequencies obtained from FEM and DSM methods at mode 3 compared to the lower modes. The validation ensures the correctness of the Wittrick–William and DSM formulation for homogeneous materials.

### 6.2. Validation of DSM Formulation—FG Beam

Further, the exactness of the FG modeling and the corresponding DSM formulation has been ensured by validating the non-dimensional natural frequencies computed for the simply supported FG beam from the developed code. The non-dimensional natural frequencies are computed for the slenderness ratio $\frac{L}{h}=20$ at different modes and for different power law indices from the developed code. The computed results are compared with results available in the literature in Table 2. The natural frequencies are in good agreement with the published results. The beam dimensions and FG material properties used for determining the frequencies are obtained from [21] as given below.

Beam dimensions: b=0.1 m, h=0.1 m.

Bottom surface (steel): $E_{b}=210\;\mathrm{GPa},\;\nu_{b}=0.31,\;\rho_{b}=7800\;\mathrm{kg}/m^{3}$.

Top surface (Al_2_O_3_): $E_{t}=390\;\mathrm{GPa},\;\nu_{t}=0.25,\;\rho_{t}=3960\;\mathrm{kg}/m^{3}$.

The non-dimensional natural frequencies are computed as

$$\lambda_{i}=\frac{\omega_{i}L^{2}}{h}\sqrt{\frac{\rho_{b}}{E_{b}}}$$

Thus, the dynamic stiffness matrix formulation presented in this work can be used to carry out a free vibration analysis of the FG rotor bearing system, and the Wittrick–William algorithm can be used to determine the modal frequencies from the global dynamic stiffness matrix of the FG rotor system.

### 6.3. DSM Method Application to Thermally Loaded FG Rotor System

In this section, the DSM approach has been demonstrated for an FG rotor bearing system (shown in Figure 7) subjected to thermal gradients with proper validations. The FG rotor is divided into four rotor elements with a total of five nodes. The FG rotor is made up of FGM, which is composed of metal and ceramic, as mentioned earlier in the paper in the material modeling section. The metal and ceramic materials considered for the material modeling of the FG rotor are stainless steel and zirconium dioxide, respectively. The temperature dependent coefficients for the material properties of stainless steel and zirconium dioxide are obtained from [18] and are tabulated in Table 3. The geometrical dimensions of the FG rotor and the properties of disk and bearings used in the present study are:

Rotor dimensions: L=0.5 m, d=0.02 m.

Bearing stiffness: $K_{b}=10^{5}\;N/m$.

Disk Properties: $M_{d}=2\;\mathrm{kg},\;I_{d}=0.0012\;\mathrm{kg}\cdot m^{2},\;I_{p}=0.0024\;\mathrm{kg}\cdot m^{2}$

The first three modes of the forward and backward whirl frequencies of the FG rotor bearing system subjected to temperature gradients at the rotor spin speed 4000 rpm and inner temperature $T_{i}=300\;K$ has been computed using the dynamic stiffness method for different power law indices and temperature gradients. The computed whirl frequencies of the FG rotor bearing system subjected to temperature gradients havebeen compared with the FEM results available in [18]. The comparison between the results from the DSM method in the paper and FEM in the literature for temperature gradients at Δ*T* = 0 K, 300 K and 600 K are presented in Table 4, Table 5 and Table 6.

The whirl frequencies computed from the DSM method are in good agreement with FEM results obtained from the literature. The FG rotor has been divided into ten finite elements to obtain the required convergence of the results using FEM in the literature. However, the results obtained from the DSM method have been found to be independent ofthe number of elements considered in the analysis, which exemplifies the exactness and computational efficiency of the DSM method.

## 7. Conclusions

The dynamic stiffness matrix formulation has been developed for the first time for the free vibration analysis of a (FG) rotor bearing system subjected to temperature gradients and to investigate its application to FG rotors. The translational and rotational equilibrium equations have been obtained by applying the equilibrium conditions to the expressions of various loads acting on the FG rotor elements. The governing equations of motion for FG rotor elements have been derived from the equilibrium equations, and the dynamic stiffness matrix has been developed for the same. The Wittrick–William algorithm has been employed as a root searching technique to compute the natural frequencies from the global DS matrix.

The dynamic stiffness matrix formulation code has been thus developed to carry out the free vibration analysis of FG rotor bearing systems. The correctness of the dynamic stiffness matrix formulation has been ensured with the validation of the results obtained from the DSM code, with the results available in the literature. The natural whirl frequencies of the FG rotor bearing system subjected to temperature gradient have been obtained from the DSM code and compared with the FEM results available in the literature. The exactness of the DSM method has been exemplified as the results obtained from the method have been found to be independent of the number of the FG rotor elements considered, which saves a significant amount of computational time.

The authors’ believe that the current article is a step in the right direction in the accurate analysis of complex systems made up of FGMs in the broader view of applications in engineering. The developed DSM method can be used to study the vibration behavior of systems used in various applications, such as micro/nano applications and so on, re-modeled using functionally graded material to investigate its possible application in those systems.

## Figures and Tables

**Table 1:** Natural frequencies of steel rotor bearing system.

Mode	Natural Frequencies (rad/s)		
	Present	FEM [28]	Error%
1	152.4	152.5	0.065
2	597.9	598.2	0.050
3	1728.2	1733.6	0.312

**Table 2:** Non-dimension natural frequencies of simply supported FG beam at L/h = 20.

Mode	k =0.1		k = 0.5		k = 1		k = 5	
	Present	[21]	Present	[21]	Present	[21]	Present	[21]
1	5.0664	5.0613	4.2971	4.2943	3.9061	3.9058	3.3035	3.3032
2	20.0268	20.0040	16.9857	16.9720	15.4375	15.4330	13.0463	13.0420
3	44.2153	44.1560	37.4997	37.4600	34.0734	34.0520	28.7623	28.7430
4	76.6660	76.5420	65.0189	64.9280	59.0597	58.9970	49.7804	49.7250

**Table 3:** Temperature coefficients of metal and ceramic material properties.

Properties	Material	*P*_0_	*P*_−1_	*P*_1_	*P*_2_	*P*_3_
*E* (Pa)	SS	201.04 × 10^9^	0	+3.079 × 10^−4^	−6.534 × 10^−7^	0
	ZrO_2_	244.27 × 10^9^	0	−1.371 × 10^−3^	+1.214 × 10^−6^	−3.681 × 10^−10^
*ν*	SS	0.3262	0	−2.002 × 10^−4^	+3.797 × 10^−7^	0
	ZrO_2_	0.2882	0	+1.133 × 10^−4^	0	0
*K* (W/m K)	SS	15.739	0	−1.264 × 10^−3^	+2.092 × 10^−6^	−7.223 × 10^−10^
	ZrO_2_	1.700	0	+1.276 × 10^−4^	+6.648 × 10^−8^	0
*ρ* (kg/m^3^)	SS	8166	0	0	0	0
	ZrO_2_	5700	0	0	0	0

**Table 4:** Whirl frequencies (in Hz) of FG rotor bearing system at Δ*T* = 0 K, Ω = 4000 rpm.

Mode	k = 0.5		k = 1		k = 5	
	Present	FEM [18]	Present	FEM [18]	Present	FEM [18]
1BW	36.167	36.166	35.980	35.979	35.531	35.530
1FW	36.168	36.169	35.981	35.981	35.533	35.533
2BW	115.473	115.421	112.845	112.794	106.266	106.213
2FW	123.916	123.974	120.866	120.924	113.274	113.333
3BW	298.710	298.593	294.381	294.259	286.371	286.243
3FW	298.999	299.121	294.668	294.785	286.666	286.784

**Table 5:** Whirl frequencies (in Hz) of FG rotor bearing system at Δ*T* = 300 K, Ω = 4000 rpm.

Mode	k = 0.5		k = 1		k = 5	
	Present	FEM [18]	Present	FEM [18]	Present	FEM [18]
1BW	35.386	35.384	35.309	35.307	35.129	35.128
1FW	35.388	35.387	35.311	35.310	35.131	35.131
2BW	115.061	115.009	112.505	112.453	106.082	106.028
2FW	123.669	123.727	120.660	120.717	113.159	113.217
3BW	279.719	279.577	277.548	277.400	275.020	274.872
3FW	280.007	280.103	277.834	277.924	275.314	275.413

**Table 6:** Whirl frequencies (in Hz) of FG rotor bearing system at Δ*T* = 600 K, Ω = 4000 rpm.

Mode	k = 0.5		k = 1		k = 5	
	Present	FEM [18]	Present	FEM [18]	Present	FEM [18]
1BW	34.870	34.868	34.837	34.834	34.723	34.723
1FW	34.872	34.872	34.839	34.838	34.725	34.726
2BW	114.774	114.722	112.253	112.201	105.889	105.836
2FW	123.498	123.555	120.508	120.564	113.039	113.097
3BW	269.173	269.020	267.446	267.286	264.933	264.812
3FW	269.459	269.545	267.731	267.809	265.227	265.352

## Data Availability

Not applicable.

## Abbreviations, Symbols and Notations

FGM	Functionally graded materials
FG	Functionally graded
DSM	Dynamic stiffness matrix
DS	Dynamic stiffness
NLTD	Non-linear temperature distribution
TBT	Timoshenko beam theory
*A*	cross-sectional area
*Pm*	material properties of metal
*Pc*	material properties of ceramic
*Vm*	volume fraction of metal
*Vc*	volume fraction of ceramic
*k*	power law index
*K*	thermal conductivity
$R_{i},R_{o}$	inner and outer radius of the FG rotor
$T_{i},T_{o}$	temperature at inner and outer surface of the FG rotor
$K_{i},K_{o}$	thermal conductivity at inner and outer surface of the FG rotor
(*x*,*y*,*s*)	coordinate system
*u*, *v*	lateral deflections in (*x*,*s*) and (*y*,*s*) planes, respectively
$\theta_{x},\theta_{y}$	bending slopes in (*y*,*s*) and (*x*,*s*) planes, respectively,
$q_{x},q_{y}$	distributed transverse force (*x*,*s*) and (*y*,*s*) planes, respectively, based on translation inertia
$Q_{x},Q_{y}$	distributed moments in (*y*,*s*) and (*x*,*s*) planes, respectively, based on rotation inertia and gyroscopic moments
$T_{x},T_{y}$	shear forces in (*x*,*s*) and (*y*,*s*) planes, respectively, based on transverse shear effects
$M_{x},M_{y}$	bending moments in (*y*,*s*) and (*x*,*s*) planes, respectively
*E*	Young’s modulus
ν	Poisson’s ratio
*G*	shear modulus
κ	shear factor
ρ	density
$I_{d}$	diametric mass moment of inertia of the disk
$I_{p}$	polar mass moment of inertia of the disk
*d*	rotor diameter
*L*	rotor or beam length
*l*	element length
R	radial distance

## References

[1] Chen Y.-H. (1987). General dynamic-stiffness matrix of a timoshenko beam for transverse vibrations. Earthq. Eng. Struct. Dyn..

[2] Curti G., Raffa F.A., Vatta F. (1991). The Dynamic Stiffness Matrix Method in the Analysis of Rotating Systems. Tribol. Trans..

[3] Curti G., Raffa F.A., Vatta F. (1992). An analytical approach to the dynamics of rotating shafts. Meccanica.

[4] Banerjee J.R. (1997). Dynamic stiffness formulation for structural elements: A general approach. Comp. Struct..

[5] Cheung Y.K., Wanji C. (1995). Refined nine-parameter triangular thin plate bending element by using refined direct stiffness method. Int. J. Numer. Methods Eng..

[6] Rafezy B., Howson W.P. (2006). Exact dynamic stiffness matrix for a thin-walled beam of doubly asymmetric cross-section filled with shear sensitive material. Int. J. Numer. Methods Eng..

[7] Wittrick W.H., Williams F.W. (1971). A General Algorithm for Computing Natural Frequencies of Elastic Structures. Q. J. Mech. Appl. Math..

[8] Williams F.W., Howson W.P., Watson A. (2004). Application of the Wittrick—Williams algorithm to the Sturm—Liouville problem on homogeneous trees: A structural mechanics analogy. Proc. R. Soc. A Math. Phys. Eng. Sci..

[9] Han F., Dan D., Cheng W., Jubao Z. (2018). An improved Wittrick-Williams algorithm for beam-type structures. Compos. Struct..

[10] Aydogdu M., Taskin V. (2007). Free vibration analysis of functionally graded beams with simply supported edges. Mater. Des..

[11] Xiang H., Yang J. (2008). Free and forced vibration of a laminated FGM Timoshenko beam of variable thickness under heat conduction. Compos. Part B Eng..

[12] Alshorbagy A.E., Eltaher M., Mahmoud F. (2010). Free vibration characteristics of a functionally graded beam by finite element method. Appl. Math. Model..

[13] Şimşek M., Reddy J.N. (2013). Bending and vibration of functionally graded microbeams using a new higher order beam theory and the modified couple stress theory. Int. J. Mech. Sci..

[14] Celebi K., Tutuncu N. (2014). Free vibration analysis of functionally graded beams using an exact plane elasticity approach. Proc. Inst. Mech. Eng. Part C J. Mech. Eng. Sci..

[15] Gayen D., Roy T. (2014). Finite element based vibration analysis of functionally graded spinning shaft system. Proc. Inst. Mech. Eng. Part C J. Mech. Eng. Sci..

[16] Rao D.K., Roy T. (2016). Vibration Analysis of Functionally Graded Rotating Shaft System. Procedia Eng..

[17] Gayen D., Chakraborty D., Tiwari R. (2016). Whirl frequencies and critical speeds of a rotor-bearing system with a cracked functionally graded shaft—Finite element analysis. Eur. J. Mech./A Solids.

[18] Sathujoda P., Batchu A., Obalareddy B., Canale G., Maligno A., Citarella R. (2020). Free Vibration Analysis of a Thermally Loaded Porous Functionally Graded Rotor–Bearing System. Appl. Sci..

[19] Sathujoda P., Obalareddy B., Batchu A., Canale G., Maligno A., Citarella R. (2020). Effect of Corrosion on the Natural and Whirl Frequencies of a Functionally Graded Rotor-Bearing System Subjected to Thermal Gradients. Materials.

[20] Su H., Banerjee J.R., Cheung C.W. (2013). Dynamic stiffness formulation and free vibration analysis of functionally graded beams. Compos. Struct..

[21] Su H., Banerjee J. (2015). Development of dynamic stiffness method for free vibration of functionally graded Timoshenko beams. Comput. Struct..

[22] Banerjee J.R., Ananthapuvirajah A. (2018). Free vibration of functionally graded beams and frameworks using the dynamic stiffness method. J. Sound Vib..

[23] Hao Y., Gao M., Gong J. (2022). Parametric Random Vibration Analysis of an Axially Moving Laminated Shape Memory Alloy Beam Based on Monte Carlo Simulation. Materials.

[24] Akgöz B., Civalek Ö. (2015). A novel microstructure-dependent shear deformable beam model. Int. J. Mech. Sci..

[25] Akbaş Ş., Ersoy H., Akgöz B., Civalek Ö. (2021). Dynamic Analysis of a Fiber-Reinforced Composite Beam under a Moving Load by the Ritz Method. Mathematics.

[26] Lanhe W. (2004). Thermal buckling of a simply supported moderately thick rectangular FGM plate. Compos. Struct..

[27] Reddy J.N., Chin C.D. (1998). Thermomechanical Analysis of Functionally Graded Cylinders and Plates. J. Therm. Stress..

[28] Sekhar A.S., Prasad P.B. (1997). Dynamic Analysis of a Rotor System Considering a Slant Crack in the Shaft. J. Sound Vib..

